# The Effects of the Drought of 1921 on Water Supply

**Published:** 1922-06

**Authors:** 


### THE EFFECTS OF THE DROUGHT OF 1921 ON WATER SUPPLY.

It may seem a far cry from the famine in Russia to the water supply of some remote village in this country, and yet to the public health officer and sanitarian the connection between the two has been evident for some time. Famine gives rise to disease, and the recent westward march of disease, in particular cholera, reported by the European Health Commission sitting in Warsaw, must remind us that special measures should be taken to prevent the occurrence of epidemics here. From an international standpoint steps are already being taken to fight the famine, and from a national standpoint we must be prepared to fight the disease should the necessity arise. Our first line of defence is, of course, the sea, our second line the vigilance of Port Sanitary Authorities, and our third line the protection of our water supplies in order to prevent the spread of water-borne disease.

So long ago as September last the Ministry of Health recognised that the prolonged drought and the prevalence of epidemics in foreign countries had increased the risk of danger to health from waterborne disease, and issued a circular (241) to Local Authorities, urging them to take every precaution to ensure the purity of the water supplies and recommending chlorination in the case of supplies which had become polluted or which were suspected of spreading disease. In March a further circular (288) was issued by the Ministry to Local Authorities and Water Companies, pointing out that a shortage of water still prevails in many parts of the country and that sources which have hitherto been satisfactory may, in consequence of the drought, become liable to pollution. The circular did not call attention to the continued prevalence of epidemics in some foreign countries, nor point out to Water Authorities the necessity for continued vigilance even in those cases where the effects of the drought would appear to be past in so far as a shortage of water is concerned.

In considering the effect of the drought upon water supplies it is necessary to bear in mind the possible effect both on the quantity and on the quality of the water. Not only has the low rainfall of 1921, amounting in many cases, especially in the south of England, to less than 50 per cent. of the average, resulted in a diminished yield from upland sources, but the attendant excessive sunshine has increased the evaporation by some 20 or 30 per cent., so that the amount of water percolating to underground sources has been diminished to an even greater extent than that from upland or surface sources. At the Rothamsted Experimental Station at Harpenden the following table shows the rainfall and percolation for 1921 as compared with the averages for 50 years.

| Rainfall. | Average for 50 years. Ins. | 1921. Ins. |
|---|---|---|
| 1/1000 acre gauge ... | 28·692 | 16·093 |
| Percolation through — | | |
| 20 in. soil ... ... | 14·834 | 5·766 |
| 40 in. soil ... ... | 15·482 | 5·981 |
| 60 in. soil ... ... | 14·659 | 5·479 |

The soil at Rothamsted is a rather heavy loam, with a reddish subsoil over chalk.

In considering rainfall statistics, and especially in comparing annual records, the calendar year has many disadvantages, and a seasonal year from July to June yields a much better picture of what has actually taken place from a water supply point of view. It is upon the rain which falls between October and March that deep-seated sources are dependent for supplies, and it is, therefore, little short of absurd to break annual records in the middle of this period. As an illustration, the rain which fell in the Thames Valley during the first three months of the present year was above the average, being well above in January and February and below in March. The rainfall for the six months from October, 1921, to March, 1922, however, has been 1·5 inches (or about 10 per cent.) below the average, and underground sources have been very inadequately replenished, so that the danger of a water shortage during the coming summer is very real.

As regards the quantity of water, the drought of last year affected seriously some 200 water authorities, and in about 50 cases the available supply was less than half the normal supply. Many of these authorities have not yet attained to the normal supply, and they are consequently taking steps to secure legislation which will place at their disposal an additional supply. In some cases additional supplies can be obtained without legislation.

It is most important not only that a community should be supplied with an adequate amount of water, but that the water should be pure. The obligations imposed upon local authorities by the Public Health Acts are very definite in this respect, and the drought has rendered liable to pollution supplies which have hitherto proved satisfactory. In the first place, any minimal amount of pollution which is normally present in the raw water is doubled in concentration if the volume of the raw water is halved. This means that the purifying effect of a large volume of water upon existing pollution is largely lost, with the result that an extra burden is thrown upon the purification works of the water supplying authority. This consideration will apply largely to surface water supplies.

In the second place, in the case of underground sources of supply, cracks in the parched earth may have been formed in the neighbourhood of wells, and through these polluting matter may gain access to the water. In the case of chalk wells, the pumping even of normal quantities of water may so lower the water level that some underground current of water may be reversed, and result in the flowing into the supply of some polluted water. This consideration also applies to wells near the sea coast, in which case an influx of salt water may occur. These possibilities indicate how necessary it is that local authorities and those responsible for supplying water to communities should be on the *qui vive* for any alteration in the quality of the water supplied, as the danger of such an alteration has been increased so very largely as a result of the recent drought. The importance of having the quality of the water supplied under scientific control cannot be too highly emphasised, and the example set by the Metropolitan Water Board should be maintained and followed in a larger number of cases.

Finally, the drought, like the Great War, opened our eyes to the advantages of scientific control. Both gave an impetus to chlorination as a safeguard of the purity of water supply, and it is devoutly to be hoped that the lessons learned will not readily be forgotten.

---

Several examinations were held in London on April 21 and 22 by the Royal Sanitary Institute, and certificates have now been awarded as follows:—

| Examination. | Candidates examined. | | Certificates awarded. |
|---|---|---|---|
| Sanitary Science as applied to Buildings and Public Works ... ... ... | 19 | ... | 2 |
| Tropical Hygiene for Sanitary Inspectors ... ... | 3 | ... | 1 |
| Women Health Visitors and School Nurses ... ... | 57 | ... | 32 |
| Maternity and Child Welfare Workers ... ... | 6 | ... | 2 |
| School Hygiene ... ... | 37 | ... | 34 |
| Sanitary Inspectors ... | 48 | ... | 24 |

